# Burden of fractures in France: incidence and severity by age, gender, and site in 2016

**DOI:** 10.1007/s00264-020-04492-2

**Published:** 2020-02-08

**Authors:** Benjamin Bouyer, Fanny Leroy, Jérémie Rudant, Alain Weill, Joël Coste

**Affiliations:** 1 Service d’Orthopédie, Hôpital Européen Georges Pompidou, 75015 Paris, France; 2 Université de Paris, Paris, France; 3 Caisse Nationale de l’Assurance Maladie, Paris, France; 4 Groupement d’Intérêt Scientifique EPI-PHARE, Saint-Denis, France; 5 Unité de Biostatistique et d’Epidémiologie, Hôpital Cochin, 27 rue du Faubourg Saint Jacques, 75014 Paris, France

**Keywords:** Fractures, General population, Epidemiology, Incidence, Severity

## Abstract

**Purpose:**

Fractures are common events, but the exact incidence and severity of fractures have not been clearly determined for most anatomical sites. We estimated the incidence and severity of fractures in France regardless of the anatomical site.

**Methods:**

Observational cross-sectional study in France in 2016 based on the national health data system. All incident fractures in patients 20 years and older were included. We determined the anatomical fracture site (12 sites) and the severity using a 4-point scale (outpatient care, hospitalization, surgery, and in-hospital death).

**Results:**

We identified 562,094 incident fractures, predominantly occurring in women (319,858: 56.9%); with a mean age of 63.6 years, and an exponential increase after the age of 70 years. Distal upper limb (172,591: 30.7%), distal lower limb (84,602: 15.1%), and femoral neck (78,766: 14.0%) accounted for more than one-half of all fractures. Sex and age of onset distributions varied widely according to fracture sites, with earlier onset for distal lower limb fractures (mean age: 54.2 years) and distal upper limb fractures (mean age: 55.2 years) with a men predominance for skull fractures. Only 105,165 (18.7%) fractures were treated on an outpatient basis; 11,913 (2.1%) in-hospital deaths occurred in patients with a mean age of 79.5 years. High mortality was observed for skull (12.9%), rib (4.9%), and femoral fractures (femoral neck 4.3% and proximal lower limb 4.2%).

**Conclusion:**

We estimated the incidence of fractures in France by sex and anatomical site. We also showed that fractures remain common and serious life events, especially in older people.

**Electronic supplementary material:**

The online version of this article (10.1007/s00264-020-04492-2) contains supplementary material, which is available to authorized users.

## Introduction

Fractures are frequent events in a subject’s lifetime [1]. Osteoporotic or low-trauma fractures are the most extensively studied types of fractures and have become a worldwide subject of interest [2, 3]. The incidence of fractures increases dramatically with age in all countries [4–6].

Hip fractures have been studied in particular detail, as they are associated with a high mortality rate, but the burden of all fractures is constantly growing in all countries as a result of aging of the populations [7].

Few recent studies have addressed the nationwide global burden of fractures, taking into account fracture severity and health care utilization. Fractures, particularly low-trauma fractures, primarily hip fractures, are responsible for loss of healthy life-years or increased mortality [8, 9].

However, no nationwide general population data are available concerning the severity and consequences of fractures regardless of age, and according to the anatomical fracture sites.

The aim of this study was to estimate the incidence and severity of fractures in France in 2016 and analyze variations according to age, gender, and anatomical fracture site.

## Methods

### Source

This study was conducted using the national health data system (SNDS) which covers the entire French population (66.6 million inhabitants). Fractures were identified in two-linked databases, the French national health insurance database (DCIR) and the French hospital discharge database (*Programme de Médicalisation des Systèmes d’Information*, PMSI), by unique anonymous numbers. The DCIR allows access to reimbursed medical care or products, including outpatient drugs, medical devices, and medical procedures. The PMSI provides information about medical diagnoses and surgical procedures during the hospital stay, including death when it occurs in hospital. These databases have been used to conduct various pharmaco-epidemiological studies and Health Services Research [10] and for the assessment of several surgical procedures, including orthopaedic surgery [10–14].

### Patients

All subjects aged 20 years or older on 31 December 2015 living in France (including overseas departments) were eligible for inclusion. Patients with at least one fracture in 2016 were included.

Fractures were detected in two ways: patients requiring hospital admission, by using a list of ICD-10 diagnosis codes when the fracture was indicated in the main diagnosis, related or associated diagnosis; and patients treated on an outpatient basis, by using a list of orthopedic splinting procedure codes from the French medical classification of clinical procedures (*Classification Commune des Actes Médicaux* -CCAM). The list of codes is presented in supplementary Table 1.

To avoid duplicate (prevalent) events or treatment for previous fracture care, patients were excluded when a similar fracture was detected during the previous three months, or when an orthopaedic implant removal procedure was performed (list in supplementary Table 2).

### Anatomical fracture site

Due to the limited information recorded when the fracture is treated on an outpatient basis (no morbidity coding is performed in the ambulatory care setting in France, only procedures such as plaster casts are recorded), we defined 12 fracture site categories: skull, face, spine, pelvis, clavicle, rib, proximal upper limb, distal upper limb, femoral neck, proximal lower limb, distal lower limb, and multiple fractures when at least two sites were detected, according to ICD-10 codes or according to the type of plaster applied. Details are given in supplementary Table 3.

### Severity

Severity was classified into four levels depending on the medical consequences of the fracture: orthopedic immobilization in the outpatient setting (level 1), hospital admission without surgery (level 2), hospital admission with a surgical procedure on the fracture, (procedures were identified when a surgical procedure corresponding to the fracture was performed during the same hospitalization) (level 3), and in-hospital mortality, as recorded by the PMSI (level 4). In patients with several repeated fractures, the most severe fracture was included in the analysis.

### Statistical analysis

The total number of fractures in the overall population was reported with the estimated mean age and standard deviation (SD) of the patients concerned. The number and percentage of fractures with the estimated mean age and standard deviation of the population concerned were calculated for the following combinations of variables: gender, fracture site, severity, gender and fracture site, gender and severity, gender and fracture site, and severity.

The incidence rate per 10,000 person-years and the 95% confidence interval for each fracture site were estimated by gender and 5-year age intervals from 20 to 90 years and older. The French population for the gender-age groups on 1 January 2016 was obtained from INSEE data [10].

## Results

In the population of 49,763,610 adult patients included in this study, we identified 562,094 subjects with an incident fracture in 2016 (1.1%). These subjects had a mean age of 63.6 years (SD: 22.1), and presented a female predominance: 319,858 (56.9%). Details of the fracture distribution by gender and fracture sites are presented in Table 1. Three sites accounted for more than half of all fractures: distal upper limb (172,591, 30.7%), distal lower limb (84,602, 15.1%), and femoral neck (78,766, 14%). Skull, face, clavicle, distal upper limb, and distal lower limb fractures presented a bimodal distribution, especially in men and occurred earlier in life at a mean age of 58.2, 51.6, 52, 55.2, and 54.2 years, respectively, whereas pelvic, femoral neck, and proximal lower limb fractures occurred in older patients (mean age of 77.3, 82, and 77.6 years, respectively).

**Table 1:** Description of fractures in France in 2016 by fracture site

	Gender	*N* (%)	Mean age (SD)
All fractures	All	562,094 (100)	63.6 (22.1)
	Men	242,236 (43.1)	53.2 (21.8)
	Women	319,858 (56.9)	71.5 (18.9)
Skull	All	5986 (100)	58.2 (21.1)
	Men	4146 (69.3)	54 (20.2)
	Women	1840 (30.7)	67.5 (20)
Face	All	25,534 (100)	51.6 (24.2)
	Men	16,490 (64.6)	44.8 (21.2)
	Women	9044 (35,4)	64.1 (24.3)
Spine	All	49,783 (100)	71.9 (18.3)
	Men	20,762 (41.7)	66.1 (19.9)
	Women	29,021 (58,3)	76 (15.9)
Pelvis	All	13,675 (100)	77.3 (17.2)
	Men	3990 (29.2)	68 (20.7)
	Women	9685 (70.8)	81.2 (13.8)
Ribs	All	26,353 (100)	63.1 (18.9)
	Men	15,828 (60.1)	63.3 (18.4)
	Women	10,525 (39.9)	73.4 (17.3)
Clavicle	All	13,988 (100)	52 (21.6)
	Men	9075 (64.9)	46 (18.8)
	Women	4913 (35.1)	63.1 (22.1)
Proximal upper limb	All	33,690 (100)	69.1 (18.3)
	Men	11,329 (33.5)	59.4 (19.7)
	Women	22,361 (66.4)	73.9 (15.4)
Distal upper limb	All	172,591 (100)	55.2 (18.3)
	Men	76,756 (44.5)	43.6 (19.2)
	Women	95,835 (55.5)	64.6 (18.9)
Femoral neck	All	78,766 (100)	82 (11.7)
	Men	21,510 (27.3)	78 (14.3)
	Women	57,256 (72.7)	83.6 (10.2)
Proximal lower limb	All	16,922 (100)	77.6 (17.2)
	Men	4945 (29.2)	67.2 (21.7)
	Women	11,977 (70.8)	81.9 (12.7)
Distal lower limb	All	84,602 (100)	54.2 (19.8)
	Men	39,659 (46.9)	46.8 (17.9)
	Women	44,943 (53.1)	60.8 (19)
Multiple fractures	All	40,204 (100)	67.1 (21.8)
	Men	17,746 (44.1)	55.2 21)
	Women	22,458 (55.9)	76.5 (17.4)

Marked variations in the incidence of fractures were observed according to gender and according to fracture site with age, with a nonlinear increase with age.

**Fig. 1:**
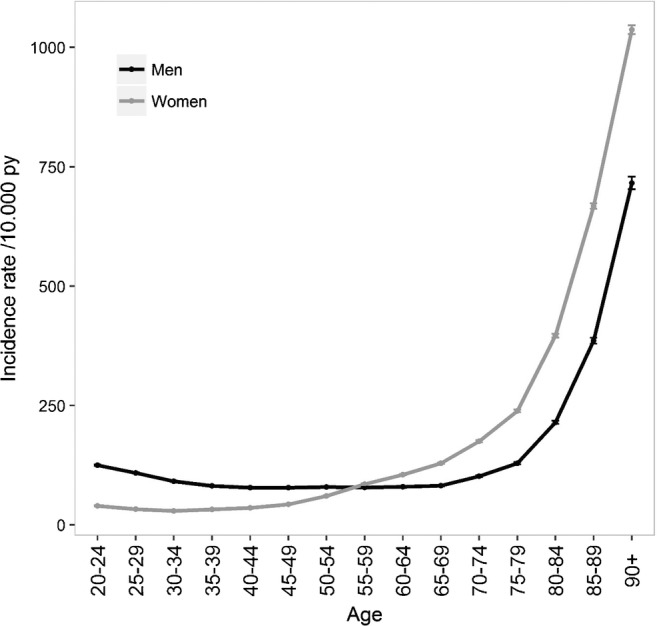
Incidence of fractures in 2016 in France for men and women

**Fig. 2:**
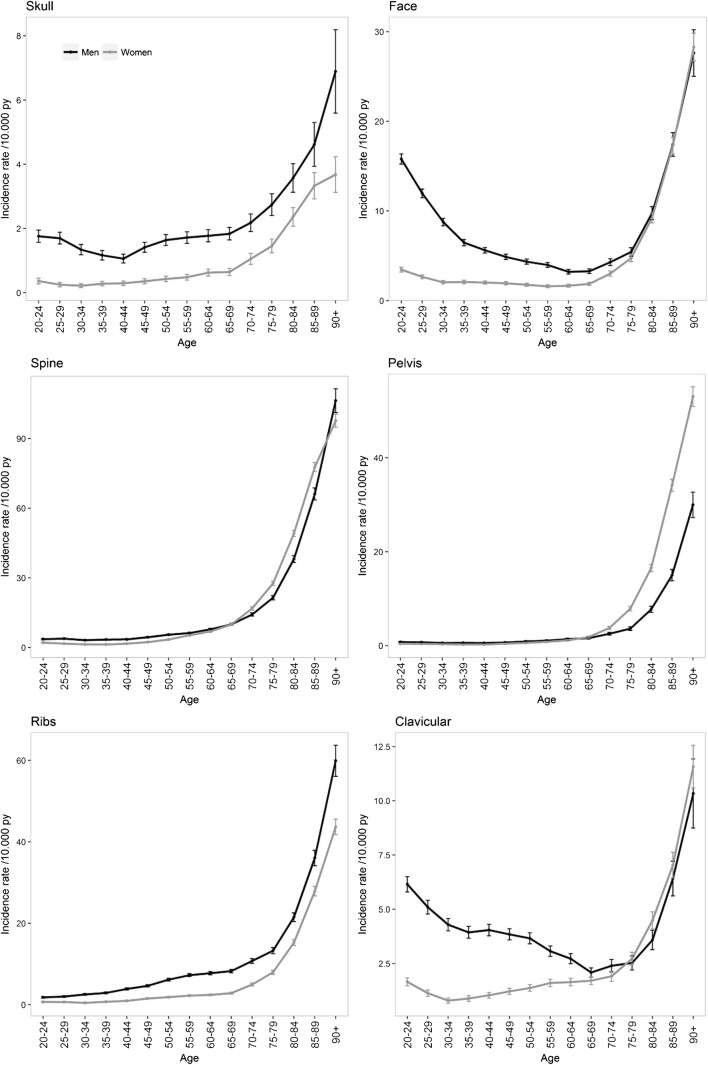
Incidence of skull, face, spine, pelvis, rib, and clavicle fractures in 2016 in France for men and women

**Fig. 3:**
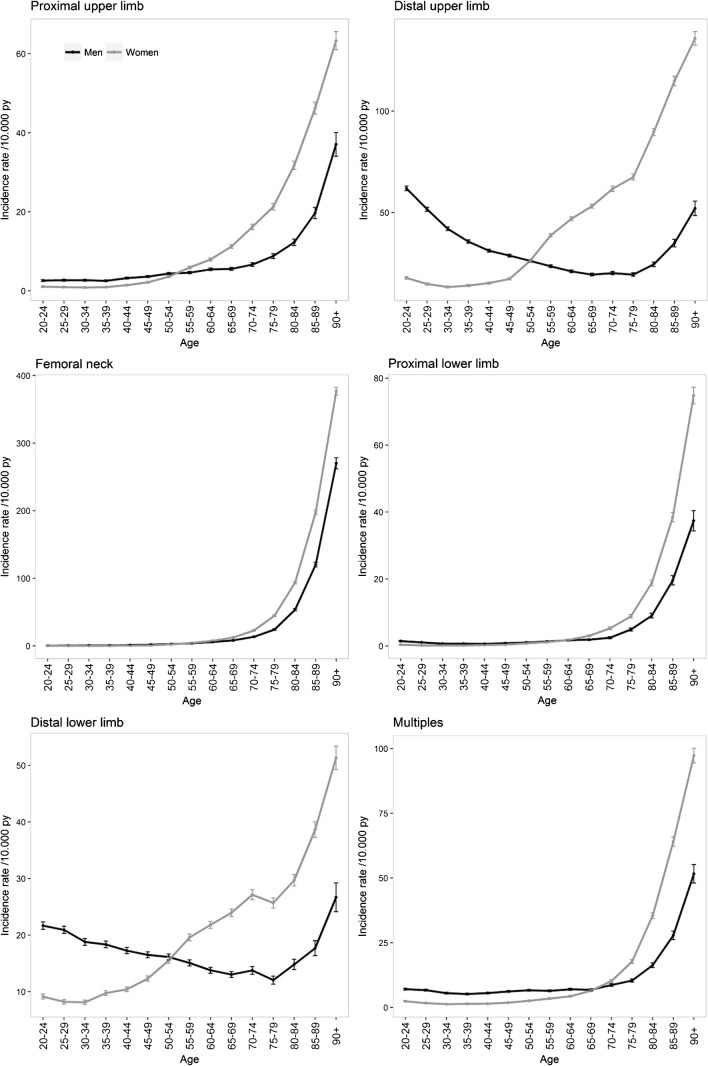
Incidence of limb and multiple fractures in 2016 in France for men and women

The burden of fractures for all sites, notably for several specific sites (clavicle, proximal and distal upper limb, distal lower limb, and face) was higher among men during the first decades of life, with a time-dependent crossover according to the site studied (mean age of 50 years for proximal and distal upper limb and lower limb fractures and 70 years for other fractures), when fractures were predominantly observed in women. Skull fractures occurred more commonly in men than in women at all ages (men accounted for 4146 of the 5986 skull fractures, 69.3%). Nevertheless, the overall pattern of the last quarter of life observed in the general population, with an exponential increase for both genders, was observed for all sites after the age of 70 years for the majority of fracture sites studied except for upper limb and distal lower limb fractures in women with an early shift of the inflection point at 55 years. Incidence curves are shown in Figs. 1, 2 and 3. Incidences of fractures by gender across age groups are presented in supplementary Tables 4 and 5.

A majority of patients with fractures required hospital admission (456,929, 81.3%) and more than half needed a surgical procedure (281,290, 50%), with a higher proportion for several sites, notably lower limb fractures: 69,030 (87.6%) and 13,213 (78.1%) patients operated on for femoral neck and proximal lower limb fractures, respectively. In contrast, 12,064 (88.2%) of the 13,675 patients with pelvic fractures were hospitalized without a surgical procedure, and 6521 (46.6%) of the 13,988 patients with clavicular fractures and 69,705 (40.4%) of the 172,591 patients with distal lower limb fractures were treated by immobilization without hospitalization.

Severity of fractures is presented in Table 2 and separately by gender in supplementary Table 6. Severity systematically increased with age for the overall population and according to fracture site from outpatient management to hospitalization and death. However, patients undergoing operations were younger than non-operated hospitalized patients. A total of 11,913 (2.1%) patients, with a mean age of 79.5 years (SD 15.9), died in hospital, including 5781 women (1.8%) and 6132 men (2.5%). The mean age of deceased patients ranged from 66 to 84 years for men and 75 to 87 years for women according the fracture site. Fractures associated with higher early mortality were skull fractures (12.9%), rib fractures (4.9%), and fractures of the femur (4.3% for femoral neck and 4.2 for proximal lower limb)

**Table 2:** Clinical severity of fractures in France in 2016 by fracture site

	Severity	*N* (%)	Mean age (SD)
All fractures	1	105,165 (18.7)	51.71 (20.51)
	2	163,726 (29.1)	70.2 (20.6)
	3	281,290 (50)	63.51 (21.78)
	4	11,913 (2.1)	79.54 (15.87)
Skull	1	NA	NA
	2	3968 (66.3)	58.38 (20.80)
	3	1249 (20.9)	50.79 (20.06)
	4	769 (12.9)	69.08 (19.04)
Face	1	417 (1.6)	40.66 (18.49)
	2	16,721 (65.5)	53.75 (24.69)
	3	8013 (31.4)	46.65 (22.14)
	4	383 (1.5)	73.92 (19.46)
Spine	1	NA	NA
	2	37,063 (74.4)	74.04 (17.74)
	3	10,899 (21.9)	63.40 (18.29)
	4	1821 (3.7)	79.01 (14.30)
Pelvis	1	NA	NA
	2	12,064 (88.2)	78.78 (15.75)
	3	1129 (8.3)	60.33 (22.51)
	4	481 (3.5)	80.74 (15.07)
Ribs	1	NA	NA
	2	22,232 (84.4)	68.62 (18.74)
	3	2835 (10.8)	60.65 (19.19)
	4	1286 (4.9)	76.30 (16.00)
Clavicle	1	6521 (46.6)	48.32 (19.80)
	2	3585 (25.6)	65.91 (21.39)
	3	3693 (26.4)	43.87 (17.63)
	4	189 (1.4)	74.07 (19.54)
Proximal upper limb	1	3002 (8.9)	54.34 (20.98)
	2	12,420 (36.9)	74.39 (17.00)
	3	17,581 (52.2)	67.42 (16.97)
	4	687 (2)	79.19 (15.37)
Distal upper limb	1	69,705 (40.4)	52.78 (21.04)
	2	12,809 (7.4)	70.59 (21.58)
	3	89,677 (52)	54.83 (20.48)
	4	400 (0.2)	79.69 (16.29)
Femoral neck	1	NA	NA
	2	6327 (8)	81.47 (12.85)
	3	69,030 (87.6)	81.90 (11.68)
	4	3409 (4.3)	85.87 (9.58)
Proximal lower limb	1	579 (3.4)	81.81 (11.97)
	2	2421 (14.3)	79.02 (15.44)
	3	13,213 (78.1)	76.91 (17.77)
	4	709 (4.2)	81.72 (14.96)
Distal lower limb	1	24,937 (29.5)	48.75 (18.26)
	2	12,839 (15.2)	67.75 (20.49)
	3	46,415 (54.8)	53.22 (18.46)
	4	411 (0.5)	76.73 (18.45)
Multiple fractures	1	NA	NA
	2	21,280 (52.9)	70.59 (20.51)
	3	17,556 (43.7)	62.18 (22.44)
	4	1368 (3.4)	75.17 (19.68)

*NA* not applicable

## Discussion

In this study, we evaluated the burden of fractures in the adult population (49,763,610 subjects) in France in 2016. With 562,094 events requiring medical care, representing 1.1% of the population, fractures are a major health issue. The incidence of fractures in the overall population and for each fracture site increased with age and women were more commonly affected. These events were associated with particularly significant medical consequences. A majority of patients required hospital admission, represen ting 456,929 admissions in one year. Surgery was performed in nearly two-thirds of hospitalized patients, with an in-hospital mortality of more than 2% after a fracture.

Most studies on the incidence of fractures considered only a few site-specific incidences (mainly hip, or forearm), or incidence trends of these fractures [3]. The incidence patterns according to age in both genders in this study were similar to those already reported. Bone frailty and falls are the main risk factors for fracture, and are responsible for an exponential increase of the incidence of fractures in the last decades of life, but with varying degrees according to fracture site and the population studied [15]. Moreover, these differences have tended to increase during the second half of the twentieth century, with decreased bone quality and strength in modern society [16]. In 2011, Cooper et al. published a large retrospective review of these studies for the International Osteoporosis Foundation and found similar results to those observed in our study [3]. These authors showed an increase of age-adjusted incidences of major low-trauma fractures during the second half of the twentieth century until a plateau in the 1990s. These findings have been the subject of debate, and have been partially explained, by some authors, by the use of low-trauma fracture prevention strategies, more healthy behaviors, or the development of national prevention programs in developed countries [17–19]. These recent changes emphasize the need for accurate and recent epidemiologic data, as provided by the present study.

We found significant differences between incidence patterns according to fracture site, age, and gender. Fractures occurred at an earlier age at certain sites, particularly for the forearm and distal lower limb. Some authors have proposed mechanical explanations for this difference, such as the tendency to protect oneself during a fall with outstretched arms [20]. Similarly, ankle fractures appear to be strongly associated with certain mechanical factors, including sustained physical activity particularly among teenagers, independently of osteoporosis, explaining the higher incidence of ankle fractures in younger people [21–24]. These findings have already been reported by many authors. Abrahamsen et al. recently published a study on forearm fractures in the Danish population and reported consistent results [25]. Forearm and ankle fractures have even been considered to be an alert flag for subsequent low-trauma fractures [26, 27].

In a Danish population-based study in 2011, Driessen et al. also analyzed incident fractures and showed the same incidence patterns for the various fracture sites as those observed in our study [28]. However, incidence rates in young people were much lower in our study. One of the possible explanations for this lower incidence is the lack of sensitivity of our study to detect minor fractures occurring in healthy subjects only requiring simple outpatient care, such as foot fractures reported in the article by Driessen et al.

The economic burden of certain fractures, such as hip or wrist fractures, has been addressed in population-based studies, and has been estimated to be more than $10,000 per person [4, 29, 30]. Several published studies have established a strong link between hip fractures and a high mortality in the following year, but many of these studies are now out-dated. In a recent literature review, Abrahamsen observed a wide range of one year mortality rates after hip fracture, ranging from 8.4 to 36% [31]. However, the clinical impact of fractures has not been clearly determined and reported in real-life studies. Fractures have been reported to be a frequent cause of death in France in a nationwide study conducted by Ziadé et al. in 2010. However, no study has reported the detailed clinical outcome for specific fracture sites. Our study highlights the clinical impact of fractures, as more than four out of five patients required at least one hospital admission, and one-half of patients required surgery. Mortality is also not rare. We showed an overall mortality higher than 2%, and even higher than 4% for several severe fractures: skull, ribs, and femoral fractures. The 11,913 in-hospital deaths observed in our study based on 77% of the French population therefore represent more than 2% of the 596,685 deaths observed in France in 2016. Two main factors appear to be associated with mortality: severity of the fracture, with a higher mortality associated with fractures adjacent to a vital organ (brain for skull fracture or lung for rib fractures), and the frailty of the patient appears to have a major impact, as reflected by the much higher mortality rate among older patients observed for all fracture sites and all subgroups, in line with knowledge concerning fracture-related mortality in frail people [32]. We were nevertheless surprised to observe a relatively low mortality rate after femoral neck fractures (4.33%), lower than the mortality rates published in the literature [33]. The overall mortality related to femoral neck fractures was obviously underestimated, as we were only able to measure in-hospital mortality, but it has already been shown that mortality continues to increase up to one year after a fracture [7]. However, this low mortality rate could be partially explained by better peri-operative care of femoral neck fractures over recent years. In France, Boddaert et al. demonstrated a major impact of peri-operative care, with a decrease of in-hospital mortality from 7.6 to 3% after implementation of specific geriatric care [34].

This study conducted on the French administrative database (SNDS) allows a real-life, cross-sectional, population-based study with detailed description of individual clinical outcomes, providing updated knowledge on the epidemiology and impact of fractures managed by modern healthcare in a developed country.

The results of this study are subject to the main limitations usually encountered with the use of administrative databases in general and the SNDS in particular. Firstly, the lack of precision regarding information for outpatient care prevented accurate analysis of certain details: the only way to detect out-of-hospital fractures was by means of plaster cast procedures. This method induced two biases: the risk of underestimating events not requiring a plaster cast and the risk of overestimating events when plaster casts were used for trauma without fracture. This limitation probably had only a limited impact on our results, as it essentially concerned non-severe fractures (i.e., with limited functional impairment allowing outpatient care). Lack of information concerning outpatient care also prevented us from identifying the anatomical site/bone concerned by the fracture and, for the patients concerned, the fracture site was estimated by the type of plaster cast applied. It is likely that an unknown number of fractures were misclassified. Secondly, the very nature of the French healthcare system encouraged healthcare providers to maximize the expenditure declared by means of diagnostic coding procedures in order to obtain higher levels of funding. However, this bias would have tended to modify clinical outcome towards more severe outcomes rather than produce existing fractures or surgical procedures.

In conclusion, in this study, we documented the incidence and healthcare burden of fractures in adults in France in 2016. This study provides recent exhaustive and reliable data precisely describing the burden of fractures across age. This study also confirmed that fractures remain frequent serious life events, especially in older people.

## Electronic supplementary material

ESM 1 (DOCX 61 kb)
