# Supplementary material for: Burden of fractures in France: incidence and severity by age, gender, and site in 2016
Source: Int Orthop. 2020 Feb 8;44(5):947–55. doi: 10.1007/s00264-020-04492-2 (PMC7190681; doi:10.1007/s00264-020-04492-2)
Supplement: Supplementary file 1 — (DOCX 61 kb) [file 264_2020_4492_MOESM1_ESM.docx]

Burden of fractures in France. Incidence and severity by age, gender and site in 2016

Supplementary materials

=

Supplementary table 1. List of diagnosis and procedures codes for identification of fractures and its sites

| Site | IICD-10 Diagnosis codes | Cast procedure codes from CCAM |
| --- | --- | --- |
| Skull | S02.0, S02.1, S02.7, S02.8, S02.9 | LAEA008 |
|  | S02.2, S02.3, S02.4, S02.5, S02.6, S02.7, S02.8, S02.9 | LAEA001, LAEA003, HBED009, HBED015, LAEP002, LAEP003, LAEP001, LBED001, LBED004, LBEP009, LAEA007, LAEB001, LBEP002, LBED002, LBED005, LBED006, LBED003 |
| Spine | S12.0, S12.1, S12.2, S12.7, S22.0, S22.1, S32.0, S32.7, S32.8, S12.8, S12.9, M48.5, T08 | NA |
| Pelvis | S32.1, S32.2, S32.3, S32.4, S32.5, S32.7, S32.8, S12.8, S12.9 | NAEP002, NAEP001 |
| Clavicle | S42.0 | MADP001 |
| Ribs | S22.2, S22.3, S22.4, S22.5 | NA |
| Proximal upper limb | S42.1, S42.2, S42.3, S42.7, S42.8, S42.9, M80.01, M80.11, M80.21, M80.31, M80.41, M80.51, M80.81, M80.91, M80.02, M80.12, M80.22, M80.32, M80.42, M80.52, M80.82, M80.92 | NA |
| Distal upper limb | S42.4, S52.0, S52.1, S52.2, S52.3, S52.4, S52.5, S52.6, S62.0, S62.1, S62.2, S62.3, S62.4, S62.5, S62.6, S62.7, S52.7, S52.9, S62.8, S52.8, M80.03, M80.13, M80.23, M80.33, M80.43, M80.53, M80.83, M80.93, M80.04, M80.14, M80.24, M80.34, M80.44, M80.54, M80.84, M80.94 | MZMP002, MBEP001, MBEP003, MBEB001, MAEP001 |
| Femoral Neck | S72.0, S72.1 | NA |
| Proximal lower limb | S72.2, S72.3, S72.4, S72.7, S72.8, S72.9 | NBEP002, NBEP001, NBEB001 |
| Distal lower limb | S82.0, S82.1, S82.2, S82.4, S82.5, S82.6, S82.8, S82.3, S92.0, S92.1, S92.2, S92.3, S92.4, S92.5, S92.7, S92.9, S82.7, S82.9, M80.06, M80.16, M80.26, M80.36, M80.46, M80.56, M80.86, M80.96, M80.07, M80.17, M80.27, M80.37, M80.47, M80.57, M80.87, M80.97 | NZMP008, NZMP006, NZMP014, NCEP002, NCEP001 |

CCAM: French common classification of medical procedures

NA: Not appropriate

Supplementary table 2. List of codes of implants removal procedure from CCAM

| LAGA002, LAGA003, LAGA004, LAGA005, LDGA001, LDGA002, LEGA001, LEGA002, LFGA001, LHGA004, LHGA006, LHGA007, LJGA001, LJGA002, MAGA001, MDGA002, MDGB001, NAGA001, NBGA007, NDGA003, PAGA008, PAGA009, PAGA010, PAGA011, PAGB001, PAGB002, PAGB003, PAGB004, PAGH001, PAKB001, PAMP001, , MEGA001, MEGA002, MEKA001, MEKA002, MEKA003, MEKA004, MELA001, MFGA001, MFKA001, MGGA001, MGGA002, MGKA001, NEDA002, NEGA001, NEGA002, NEGA003, NEGA004, NEGA005, NEKA001, NEKA002, NEKA003, NEKA004, NEKA005, NEKA006, NEKA007, NEKA008, NEKA009, NEKA013, NEKA015, NEKA019, NEKA022, NELA001, NELA002, NELA003, NEMA011, NFGA001, NFGA002, NFKA001, NFKA002, NFKA003, NFKA004, NFKA005, NFLA001, NFLA002, NFMA006, NGGA001, NGGA002 |
| --- |

CCAM : French common classification of medical procedures

Supplementary table 3. List of procedure codes of surgery from CCAM

| List of procedure codes |
| --- |
| AAFA006 AAFA008 AAJA001 AAJA002 AAJA003 AAJA004 AAJA005 AAJA006 AAJH001 AAJH004 ABCA002 ABCA003 ABCA004 ABCB001 ABJA002 ABJA003 ABJA004 ABJA005 ABJA006 ABJA007 ABJA008 ABMA002 ABMA003 ABSA001 ABSA002 ABSA003 ABSA004 ABSA005 ABSA006 ABSA007 ABSA011 ABSA012 ACQP002 ADCA002 ADEA001 ADEA002 ADPA001 ADPA008 ADPA011 ADPA016 ADPA020 ADPA021 AEJA002 AEJA003 AEJA004 AEJA005 AFCA002 AFCA004 AFJA001 AFJA002 AFJA003 AFJA004 AFJA005 AFJB002 AFPA001 AGMA001 AHCA002 AHCA003 AHCA004 AHCA005 AHCA006 AHCA008 AHCA009 AHCA010 AHCA011 AHCA012 AHCA013 AHCA015 AHCA016 AHCA017 AHCA018 AHCA019 AHCA021 AHCA022 AHCA023 AHEA004 AHEA006 AHEA008 AHEA009 AHEA014 AHEA016 AHFA009 AHPA001 AHPA002 AHPA003 AHPA004 AHPA005 AHPA006 AHPA008 AHPA009 AHPA010 AHPA011 AHPA012 AHPA013 AHPA016 AHPA017 AHPA018 AHPA019 AHPA020 AHPA021 AHPA022 AHPA023 AHPA024 AHPA026 AHPA027 AHPA028 AHPC001 BABA001 BACA001 BACA002 BACA003 BACA005 BACA006 BACA007 BACA008 BAEA001 BAEA002 BAFA008 BAFA015 BAMA004 BAMA010 BAMA013 BBGA001 BCCA001 BDCA001 BDCA002 BDCA003 BDCA004 BEJB002 BFPP001 BGCA002 BGGA002 BGGA003 BHGA001 BHGA002 BHGA003 BHGA006 BHMA001 BHMA002 BJCA001 BJEA002 BJMA002 BJMA005 BKGA003 BKGA005 BKJA001 BKMA001 BKMA003 CAEA002 CAFA009 CAJA002 CAMA016 CAMA022 CBLD001 CCPA001 DACA001 DAFA006 DBLA004 DBLF001 DCFA001 DCJA001 DCJB001 DEEF001 DFCA001 DFFA003 DFNF002 DGCA001 DGCA002 DGCA004 DGCA005 DGCA006 DGCA007 DGCA009 DGCA011 DGCA012 DGCA014 DGCA020 DGCA022 DGCA025 DGFA003 DGFA004 DGFA015 DGGA003 DGKA001 DGKA002 DGKA003 DGKA009 DGKA011 DGKA015 DGKA025 DGLF001 DGLF002 DGLF003 DGLF005 DHCA001 DHCA003 DHSA002 EAMA001 EAMA002 EASF012 EBCA009 EBFA002 EBFA003 EBFA006 EBFA008 EBFA009 EBFA012 EBFA015 EBFA016 EBKA001 EBSA008 EBSA010 ECCA001 ECCA002 ECCA003 ECCA004 ECCA005 ECCA007 ECCA010 ECFA002 ECFA005 ECLF003 ECLF004 ECSA003 EDCA001 EDCA002 EDCA003 EDCA004 EDCA005 EDFA001 EDFA002 EDFA007 EDFA010 EDKA002 EDKA003 EDLF004 EDLF005 EDLF006 EDLF013 EDSA003 EECA001 EECA002 EECA003 EECA005 EECA007 EECA008 EECA009 EECA010 EECA012 EEFA001 EEFA002 EEFA003 EEFA004 EEFA006 EEGA001 EEGA002 EEJF001 EEKA001 EELF002 EESA001 EFCA001 EFCA002 EFFA001 EFJF001 EFLF001 EFSA001 EGCA002 EGFA004 EGFA009 EGPA001 EHBD001 EHCA008 EHFA001 EJCA001 EJCA002 EJSA001 EJSA003 ENFA001 ENFA004 ENFA005 ENFA006 EPCA002 EPCA003 EPFA006 EQBP001 EZBA001 EZBA002 EZCA001 EZCA003 EZCA004 EZCA005 EZJF002 EZQA001 EZQH004 FCPA001 GABD001 GABD002 GAEA001 GAGD002 GAJA002 GAMA007 GAMA012 GAMA018 GAMA021 GBFA004 GDCA001 GDMA003 GECA001 GECA003 GFCA001 GFCC001 GFFA004 GFFA009 GFFA013 GFFA017 GFFA018 GFFA019 GFFA021 GFFA022 GFFA024 GFFA025 GFFC002 GGBA001 GGCA001 GGHB001 GGJA001 GGJA002 GGJA003 GGJA004 GGJB002 GGJB005 GGJC001 GGJC002 GGLB006 GGNA001 GGNC001 GGPA001 GGPA002 GHJA001 GHJA002 HAFA024 HAFA028 HAJA003 HAJA006 HAJA007 HAJA008 HAJA009 HAJA010 HAMA002 HAMA003 HAMA005 HAMA023 HAMA027 HAMA029 HASA018 HBDD007 HBDD008 HBDD014 HBED010 HBED011 HBED016 HBED019 HBED020 HBMA001 HDCA002 HDPA001 HECA001 HECA002 HECA004 HFCA003 HFCC001 HGCA002 HHCA001 HHCC001 HJCA001 HJCC001 HJCD001 HJCD002 HJFA008 HKCA003 HKCA004 HLFA019 HMFA010 HPBA001 HPJB001 JCCA003 JCMA003 JDCA003 JDCC016 JECA002 JFFA016 JHCA004 JHCA006 JHEP001 JJCA002 JLCA008 JLJA001 JLJA002 JMCA002 JMCA006 JNMD002 KCFA005 LABA001 LABA003 LABA004 LACA001 LACA002 LACA003 LACA004 LACA005 LACA006 LACA007 LACA008 LACA009 LACA010 LACA011 LACA012 LACA013 LACA014 LACA015 LACA016 LACA017 LACA018 LACA019 LACA020 LACB001 LACB002 LAEA001 LAEA003 LAEA007 LAEA008 LAEB001 LAFA005 LAFA006 LAFA008 LAFA900 LAGA001 LAGA007 LAHA001 LAHA002 LALA002 LAMA003 LAMA004 LAMA005 LAMA006 LAMA007 LAMA008 LAMA009 LAMA010 LAMA012 LAPA001 LAPA002 LAPA003 LAPA004 LAPA005 LAPA008 LAPA012 LAPA013 LAPA015 LAPA016 LARA004 LBAA001 LBBA004 LBBA006 LBCA001 LBCA002 LBCA003 LBCA004 LBCA005 LBCA006 LBCA007 LBCA008 LBCA009 LBCA010 LBCB001 LBCB002 LBDD001 LBEA001 LBFA003 LBFA004 LBFA018 LBFA020 LBFA023 LBGA005 LBGD001 LBLD002 LBLD016 LBLD024 LBPA002 LBPA006 LBPA010 LBPA012 LBPA015 LBPA022 LBPA028 LBPA038 LCJA002 LCJA003 LCJA004 LDAA001 LDAA002 LDCA001 LDCA002 LDCA003 LDCA004 LDCA005 LDCA006 LDCA007 LDCA008 LDCA009 LDCA010 LDCA011 LDCA012 LDCA013 LDDA001 LDFA002 LDFA003 LDFA004 LDFA005 LDFA009 LDFA010 LDFA012 LDHA002 LDKA900 LDPA001 LDPA003 LDPA006 LDPA007 LDPA008 LDPA009 LECA001 LECA002 LECA003 LECA004 LECA005 LECA006 LECC001 LEFA001 LEFA004 LEFA005 LEFA006 LEFA007 LEFA008 LEFA009 LEFA010 LEFA014 LEHA002 LEHC001 LEMA002 LEMA004 LEPA003 LEPA008 LEPA009 LFAA001 LFAA002 LFCA001 LFCA002 LFCA003 LFCA004 LFCA005 LFCC001 LFDA001 LFDA002 LFDA003 LFDA004 LFDA005 LFDA006 LFDA007 LFDA008 LFDA009 LFDA010 LFDA012 LFDA013 LFFA001 LFFA004 LFFA005 LFFA006 LFFA007 LFFA008 LFFA009 LFFA012 LFFA013 LFHA001 LFKA001 LFMA001 LFPA001 LFPA002 LFPA003 LGCA001 LGDA001 LGFA001 LGFA005 LHCA001 LHCA002 LHCA010 LHCA011 LHCA016 LHDA001 LHDA002 LHFA001 LHFA013 LHFA016 LHFA019 LHFA024 LHFA025 LHFA027 LHFA028 LHFA029 LHFA031 LHHA006 LHMA003 LHMA004 LHMA006 LHMA007 LHMA008 LHMA011 LHMA013 LHMA015 LHMA016 LHMH001 LHMH002 LHMH003 LHMH004 LHMH005 LHMH006 LHMH800 LHMH801 LHMH802 LHPA003 LHPA004 LHPA006 LHPA010 LJCA001 LJCA002 LJFA002 LJFA004 LJFA006 LJFA008 LJFA009 LJFA010 LJHA001 LJJA001 LJJA002 LJMA002 LJMA003 LLBA002 LLCA003 LLCA005 LLCC001 LLCC003 LLFA003 LLFA013 LLMA003 LLMA004 LLMA008 LLMC003 LMBA001 LMMA005 MACA001 MACA002 MACA003 MACA004 MACB001 MACB002 MADP001 MAFA001 MAFA002 MAFA003 MAFA004 MAFA005 MAFA006 MAPA001 MAPA002 MAPA003 MBCA001 MBCA002 MBCA003 MBCA004 MBCA005 MBCA006 MBCA007 MBCA008 MBCA009 MBCA010 MBCA011 MBCA012 MBCB001 MBCB002 MBCB003 MBCB004 MBCB005 MBFA001 MBFA002 MBGA001 MBGA002 MBMA001 MBMA002 MBPA001 MBPA002 MBPA003 MBPA004 MBPA005 MBPA006 MCAA001 MCCA001 MCCA002 MCCA003 MCCA004 MCCA005 MCCA006 MCCA007 MCCA008 MCCA009 MCCA010 MCCA011 MCCB001 MCCB002 MCCB003 MCCB004 MCCB005 MCCB006 MCCB007 MCCB008 MCCB009 MCFA001 MCFA002 MCFA003 MCFA004 MCFA005 MCFA006 MCKA002 MCMA001 MCMA002 MCPA001 MCPA002 MCPA003 MCPA004 MCPA005 MCPA006 MCPA007 MCPA009 MCPA010 MCPA011 MCPA012 MCPA013 MCPA014 MDAA001 MDCA001 MDCA002 MDCA003 MDCA004 MDCA005 MDCA006 MDCA007 MDCA008 MDCA009 MDCA010 MDCA011 MDCA012 MDCA013 MDCA014 MDCB001 MDCB002 MDCB003 MDCB004 MDCB005 MDEA001 MDEA002 MDEA003 MDFA001 MDFA002 MDFA003 MDFA004 MDFA005 MDFA006 MDGA001 MDGA003 MDGA004 MDGA005 MDHA001 MDMA001 MDPA001 MDPA002 MDPA003 MDPA004 MDPA005 MEDA001 MEEA002 MEEA003 MEEA004 MEFA001 MEFA003 MEFA004 MEFC001 MEFC002 MEJA001 MEJC001 MEKA005 MEKA006 MEKA007 MEKA008 MEKA009 MEKA010 MEMA001 MEMA006 MEMA008 MEMA009 MEMA011 MEMA012 MEMA014 MEMA015 MEMA016 MEMA017 MEMC001 MEMC002 MEMC003 MEMC004 MEMC005 MEPA001 MEPC001 MEQC001 MEQC002 MERP001 MFCA001 MFDA001 MFDA002 MFEA001 MFEA003 MFFA001 MFFA002 MFFC001 MFJA001 MFJC001 MFKA003 MFMA001 MFMA003 MFMA005 MFPA001 MFPA002 MFPA003 MFPC001 MFQC001 MFRP001 MGCA001 MGCC001 MGDA001 MGDA002 MGEA001 MGEA002 MGFA002 MGFA003 MGFA005 MGFA006 MGFC001 MGFC002 MGFC003 MGJA001 MGJC001 MGKA002 MGKA003 MGMA002 MGMA003 MGMA004 MGMA005 MGMA006 MGPA001 MGQC001 MGRP001 MHCA001 MHCA002 MHCA003 MHDA001 MHDA002 MHDA003 MHDA004 MHDA005 MHDB001 MHEA001 MHEA002 MHEA003 MHEA004 MHFA001 MHFA003 MHJA001 MHMA001 MHMA002 MHMA003 MHMA004 MHMA005 MHPA001 MHPA002 MHPA003 MHPA004 MJAA001 MJAA002 MJCA001 MJCA002 MJCA003 MJCA005 MJCA006 MJCA007 MJCA008 MJCA010 MJCA012 MJDA001 MJDC001 MJEA001 MJEA002 MJEA003 MJEA004 MJEA005 MJEA006 MJEA007 MJEA008 MJEA010 MJEA011 MJEA012 MJEA013 MJEA016 MJEA017 MJEA018 MJEA019 MJEA021 MJEC001 MJEC002 MJFA002 MJFA003 MJFA004 MJFA006 MJFA009 MJFA010 MJFA011 MJFA012 MJFA014 MJFA015 MJFA016 MJFA018 MJGA001 MJJA001 MJJA002 MJJA003 MJJA004 MJMA002 MJMA003 MJMA009 MJMA010 MJMA012 MJMA013 MJMA015 MJMA016 MJPA001 MJPA002 MJPA003 MJPA004 MJPA005 MJPA006 MJPA007 MJPA008 MJPA009 MJPA011 MJPA012 MJPA013 MJPB001 MZEA001 MZEA002 MZEA003 MZEA005 MZEA007 MZEA010 MZEA011 MZEA012 MZFA001 MZFA002 MZFA003 MZFA004 MZFA005 MZFA006 MZFA007 MZFA010 MZFA011 MZFA013 MZGA003 MZGA004 MZHA001 MZJB001 MZMA001 MZMA002 MZMA003 MZMA004 NACA001 NACA002 NACA003 NACA004 NACA005 NACB001 NAFA001 NAFA002 NAFA004 NAGA002 NAGA003 NAHA001 NAHA002 NAMA002 NAPA007 NBCA001 NBCA002 NBCA003 NBCA004 NBCA005 NBCA006 NBCA007 NBCA008 NBCA009 NBCA010 NBCA011 NBCA012 NBCA013 NBCA014 NBCA015 NBCB001 NBCB002 NBCB003 NBCB004 NBCB005 NBCB006 NBFA001 NBFA003 NBFA004 NBFA005 NBFA007 NBFA008 NBFA009 NBGA001 NBGA002 NBGA003 NBGA006 NBGA015 NBMA001 NBMA002 NBMA003 NBPA003 NBPA004 NBPA005 NBPA010 NBPA011 NBPA014 NBPA016 NBPA019 NBPA020 NCCA001 NCCA002 NCCA003 NCCA004 NCCA005 NCCA006 NCCA007 NCCA008 NCCA009 NCCA010 NCCA011 NCCA012 NCCA013 NCCA014 NCCA015 NCCA016 NCCA017 NCCA018 NCCA019 NCCB001 NCCB002 NCCB003 NCCB004 NCCB005 NCCB006 NCCB007 NCCC001 NCEA001 NCFA001 NCFA002 NCFA005 NCFA006 NCFA008 NCFA009 NCGA001 NCGA002 NCMA001 NCMA002 NCPA001 NCPA002 NCPA003 NCPA006 NCPA007 NCPA008 NCPA009 NCPA010 NCPA012 NCPA013 NCPA014 NCPA015 NCPA016 NDAA001 NDCA001 NDCA002 NDCA003 NDCA004 NDCA005 NDCA006 NDCB001 NDCB002 NDCB003 NDCB004 NDDA001 NDDC001 NDEA002 NDFA001 NDFA002 NDFA003 NDFA004 NDFA006 NDFA007 NDFA008 NDFA010 NDGA001 NDGA002 NDMA001 NDPA001 NDPA002 NDPA003 NDPA004 NDPA005 NDPA006 NDPA007 NDPA008 NDPA009 NDPA010 NDPA011 NDPA012 NDPA013 NDPA014 NEDA003 NEEA001 NEEA002 NEEA003 NEFA001 NEFA004 NEFC001 NEHA001 NEHA002 NEJA001 NEJA002 NEJA003 NEJA004 NEJB001 NEJC001 NEKA010 NEKA011 NEKA012 NEKA014 NEKA016 NEKA017 NEKA018 NEKA020 NEKA021 NEMA013 NEMA018 NEMA019 NEPA001 NEQC001 NFCA001 NFCA002 NFCA003 NFCA004 NFCA005 NFCA006 NFCC001 NFCC002 NFDA002 NFDA003 NFDA009 NFDC001 NFEA001 NFEA002 NFEC001 NFEC002 NFFA001 NFFA002 NFFA003 NFFA004 NFFC001 NFFC002 NFFC003 NFFC004 NFJA001 NFJA002 NFJC001 NFJC002 NFKA006 NFKA007 NFKA008 NFKA009 NFMA002 NFMA004 NFMA005 NFMA008 NFMA010 NFMA011 NFMA013 NFMC001 NFMC002 NFMC003 NFMC005 NFPA001 NFPA002 NFPA003 NFPA004 NFPC001 NFPC002 NFQC001 NFRP001 NGCA001 NGDA001 NGDA002 NGDA003 NGDA004 NGDC001 NGEA001 NGFA001 NGJA001 NGJA002 NGJC001 NGJC002 NGKA001 NGMA001 NGPA001 NGPA002 NGPA003 NGPC001 NGQC001 NGRP001 NHDA001 NHDA002 NHDA003 NHDA004 NHDA005 NHDA006 NHDA007 NHDA008 NHDA009 NHDA010 NHDA011 NHFA001 NHKA001 NHMA001 NHMA002 NHMA003 NHMA006 NHMA007 NHMA008 NHPA001 NHPA002 NHPA003 NHPA004 NHPA005 NHPA006 NHRP002 NJAA001 NJAA002 NJAA003 NJAA004 NJAB001 NJBA001 NJBA002 NJCA001 NJEA001 NJEA002 NJEA003 NJEA004 NJEA006 NJEA007 NJEA008 NJEA009 NJEA010 NJEA011 NJEA012 NJFA001 NJFA003 NJFA005 NJFA009 NJMA001 NJMA002 NJMA003 NJMA004 NJMA005 NJMA007 NJMB001 NJPA002 NJPA005 NJPA006 NJPA007 NJPA009 NJPA014 NJPA015 NJPA016 NJPA017 NJPA018 NJPA019 NJPA022 NJPA025 NJPA029 NJPA030 NJPA032 NJPA034 NJPA035 NZEA001 NZEA002 NZEA007 NZFA001 NZFA002 NZFA003 NZFA004 NZFA005 NZFA006 NZFA007 NZFA008 NZFA009 NZFA010 NZFA013 NZHA001 NZJB001 PACA001 PACB001 PACC001 PADA003 PAFA003 PAFA004 PAFA005 PAFA009 PAFA010 PAGA002 PAGA003 PAGA005 PAGA006 PAKB002 PAMH001 PAPA003 PCCA001 PCCA002 PCDA001 PCEA002 PCEA003 PCEA004 PCMA001 PCPA001 PCPA002 PCPA003 PCPA004 PCPA005 PCPA006 PCPB001 PDAB001 PZMA001 PZMA002 PZMA003 PZMA004 PZMA005 QAGA001 QAGA002 QAGA004 QAJA002 QAJA003 QAJA004 QAJA005 QAJA006 QAJA009 QAJA012 QAJA013 QAMA002 QAMA005 QAMA008 QAMA013 QAMA015 QCJA001 QEHA002 QZEA008 QZEA009 QZEA028 QZEA034 QZFA009 QZFA020 QZFA023 QZFA027 QZFA029 QZFA032 QZFA038 QZFA039 QZGA003 QZGA006 QZGA007 QZJA001 QZJA002 QZJA009 QZJA011 QZJA012 QZJA013 QZJA015 QZJA016 QZJA017 QZJA021 QZJA022 QZJA023 QZJB001 QZJB002 QZMA001 QZMA002 QZMA003 QZMA004 QZMA005 QZMA007 QZMA009 QZMA010 QZMP001 QZPA008 |

CCAM : French common classification of medical procedures

Supplementary table 4. incidence of fractures by site for men (incidence rate per 10000 patients years with 95 % confidence interval)

| Age | All Fractures | Skull | Face | Spine | Pelvis | Ribs | Clavicle |
| --- | --- | --- | --- | --- | --- | --- | --- |
| 20-24 | 125 (123-126) | 1.76 (1.57-1.95) | 15.80 (15.23-16.37) | 3.59 (3.32-3.86) | 0.78 (0.66-0.91) | 1.80 (1.61-1.99) | 6.15 (5.80-6.50) |
| 25-29 | 109 (107-110) | 1.70 (1.52-1.88) | 11.97 (11.48-12.45) | 3.79 (3.52-4.06) | 0.73 (0.61-0.85) | 2.02 (1.82-2.22) | 5.10 (4.78-5.42) |
| 30-34 | 91 (90-92) | 1.34 (1.18-1.50) | 8.77 (8.36-9.18) | 3.14 (2.89-3.38) | 0.57 (0.47-0.67) | 2.53 (2.32-2.75) | 4.29 (4.01-4.58) |
| 35-39 | 82 (80-83) | 1.17 (1.02-1.32) | 6.46 (6.11-6.81) | 3.40 (3.14-3.65) | 0.59 (0.48-0.70) | 2.89 (2.66-3.12) | 3.94 (3.66-4.21) |
| 40-44 | 78 (77-79) | 1.06 (0.93-1.20) | 5.62 (5.31-5.93) | 3.51 (3.27-3.76) | 0.58 (0.48-0.68) | 3.87 (3.61-4.12) | 4.04 (3.78-4.31) |
| 45-49 | 78 (76-79) | 1.41 (1.26-1.57) | 4.88 (4.59-5.17) | 4.42 (4.14-4.69) | 0.68 (0.57-0.78) | 4.66 (4.37-4.94) | 3.84 (3.59-4.10) |
| 50-54 | 79 (78-80) | 1.64 (1.47-1.81) | 4.35 (4.07-4.62) | 5.53 (5.22-5.84) | 0.89 (0.76-1.01) | 6.16 (5.83-6.48) | 3.66 (3.41-3.91) |
| 55-59 | 78 (77-79) | 1.72 (1.54-1.90) | 3.98 (3.70-4.25) | 6.20 (5.86-6.54) | 1.08 (0.94-1.23) | 7.25 (6.88-7.62) | 3.07 (2.83-3.31) |
| 60-64 | 79 (78-81) | 1.77 (1.58-1.96) | 3.21 (2.96-3.47) | 7.85 (7.46-8.25) | 1.40 (1.23-1.56) | 7.74 (7.35-8.14) | 2.72 (2.49-2.96) |
| 65-69 | 82 (81-83) | 1.84 (1.64-2.03) | 3.27 (3.01-3.53) | 9.92 (9.47-10.38) | 1.59 (1.41-1.77) | 8.25 (7.83-8.66) | 2.09 (1.88-2.30) |
| 70-74 | 101 (100-103) | 2.18 (1.91-2.45) | 4.29 (3.91-4.67) | 14.21 (13.52-14.90) | 2.55 (2.25-2.84) | 10.74 (10.14-11.34) | 2.40 (2.11-2.68) |
| 75-79 | 129 (126-131) | 2.74 (2.40-3.08) | 5.43 (4.95-5.91) | 21.43 (20.48-22.37) | 3.63 (3.24-4.02) | 13.27 (12.52-14.01) | 2.53 (2.20-2.85) |
| 80-84 | 214 (211-218) | 3.57 (3.13-4.02) | 9.75 (9.02-10.49) | 38.07 (36.62-39.53) | 7.72 (7.07-8.38) | 21.49 (20.40-22.58) | 3.59 (3.14-4.03) |
| 85-89 | 385 (379-392) | 4.62 (3.94-5.30) | 17.42 (16.10-18.74) | 66.13 (63.56-68.71) | 15.02 (13.79-16.25) | 36.04 (34.14-37.94) | 6.42 (5.61-7.22) |
| 90+ | 716 (703-729) | 6.89 (5.59-8.19) | 27.63 (25.03-30.23) | 106.30 (101.20-111.41) | 29.99 (27.28-32.70) | 59.92 (56.08-63.75) | 10.34 (8.75-11.93) |

Supplementary table 4. incidence of fractures by site for men (incidence rate per 10000 patients years with 95 % confidence interval) (continued)

| Age | Proximal upper limb | Distal upper limb | Femoral Neck | Proximal lower limb | Distal lower limb | Multiple fractures |
| --- | --- | --- | --- | --- | --- | --- |
| 20-24 | 2.60 (2.38-2.83) | 61.87 (60.75-62.99) | 0.33 (0.25-0.42) | 1.44 (1.27-1.61) | 21.67 (21.01-22.33) | 7.06 (6.69-7.44) |
| 25-29 | 2.69 (2.46-2.92) | 51.58 (50.57-52.59) | 0.44 (0.35-0.53) | 1.06 (0.92-1.21) | 20.92 (20.28-21.56) | 6.67 (6.31-7.03) |
| 30-34 | 2.68 (2.45-2.90) | 41.96 (41.06-42.85) | 0.60 (0.50-0.71) | 0.68 (0.57-0.80) | 18.80 (18.20-19.40) | 5.53 (5.21-5.86) |
| 35-39 | 2.54 (2.33-2.76) | 35.63 (34.81-36.45) | 0.72 (0.60-0.84) | 0.69 (0.58-0.81) | 18.35 (17.76-18.93) | 5.18 (4.87-5.50) |
| 40-44 | 3.21 (2.98-3.45) | 31.11 (30.37-31.84) | 1.24 (1.09-1.39) | 0.62 (0.52-0.72) | 17.25 (16.71-17.80) | 5.57 (5.26-5.89) |
| 45-49 | 3.62 (3.37-3.87) | 28.71 (28.01-29.42) | 1.83 (1.65-2.01) | 0.82 (0.70-0.94) | 16.50 (15.97-17.03) | 6.19 (5.86-6.51) |
| 50-54 | 4.39 (4.11-4.67) | 26.10 (25.42-26.77) | 2.76 (2.54-2.98) | 1.04 (0.91-1.18) | 16.13 (15.60-16.66) | 6.64 (6.30-6.98) |
| 55-59 | 4.62 (4.33-4.91) | 23.42 (22.76-24.09) | 4.00 (3.73-4.28) | 1.32 (1.16-1.47) | 15.08 (14.55-15.61) | 6.44 (6.09-6.79) |
| 60-64 | 5.45 (5.12-5.78) | 20.92 (20.27-21.57) | 5.92 (5.58-6.27) | 1.72 (1.53-1.90) | 13.77 (13.24-14.29) | 7.00 (6.63-7.38) |
| 65-69 | 5.56 (5.22-5.90) | 19.35 (18.71-19.98) | 8.25 (7.83-8.66) | 1.86 (1.66-2.06) | 13.03 (12.51-13.55) | 6.81 (6.44-7.19) |
| 70-74 | 6.66 (6.19-7.13) | 20.06 (19.24-20.89) | 13.48 (12.81-14.16) | 2.45 (2.16-2.74) | 13.76 (13.08-14.44) | 8.67 (8.13-9.21) |
| 75-79 | 8.80 (8.20-9.41) | 19.33 (18.43-20.22) | 24.23 (23.22-25.23) | 4.91 (4.46-5.36) | 12.03 (11.32-12.74) | 10.41 (9.75-11.06) |
| 80-84 | 12.26 (11.44-13.08) | 24.39 (23.23-25.55) | 53.49 (51.77-55.21) | 9.05 (8.34-9.75) | 14.81 (13.90-15.71) | 16.28 (15.33-17.23) |
| 85-89 | 19.69 (18.28-21.09) | 34.84 (32.97-36.71) | 120.19 (116.72-123.66) | 19.58 (18.18-20.99) | 17.68 (16.35-19.01) | 27.83 (26.16-29.49) |
| 90+ | 37.07 (34.06-40.09) | 52.00 (48.43-55.57) | 270.10 (261.96-278.24) | 37.39 (34.36-40.42) | 26.67 (24.11-29.23) | 51.62 (48.06-55.18) |

Supplementary table 5. incidence of fractures by site for women (incidence rate per 10000 patients years with 95 % confidence interval)

| Age | All fractures | Skull | Face | Spine | Pelvis | Ribs | Clavicle |
| --- | --- | --- | --- | --- | --- | --- | --- |
| 20-24 | 39 (39-40) | 0.36 (0.28-0.45) | 3.46 (3.19-3.72) | 2.11 (1.90-2.32) | 0.41 (0.32-0.50) | 0.68 (0.56-0.80) | 1.66 (1.47-1.84) |
| 25-29 | 33 (32-33) | 0.25 (0.18-0.32) | 2.65 (2.42-2.87) | 1.65 (1.47-1.82) | 0.37 (0.28-0.45) | 0.67 (0.56-0.79) | 1.13 (0.99-1.28) |
| 30-34 | 29 (28-30) | 0.22 (0.15-0.28) | 2.04 (1.84-2.23) | 1.34 (1.18-1.49) | 0.32 (0.24-0.39) | 0.45 (0.36-0.54) | 0.79 (0.67-0.91) |
| 35-39 | 32 (31-33) | 0.28 (0.21-0.35) | 2.07 (1.88-2.27) | 1.28 (1.13-1.44) | 0.26 (0.19-0.33) | 0.73 (0.62-0.85) | 0.89 (0.76-1.01) |
| 40-44 | 35 (35-36) | 0.29 (0.22-0.36) | 2.01 (1.83-2.20) | 1.66 (1.49-1.83) | 0.26 (0.19-0.32) | 0.94 (0.82-1.07) | 1.04 (0.90-1.17) |
| 45-49 | 43 (42-43) | 0.36 (0.28-0.43) | 1.94 (1.76-2.12) | 2.29 (2.10-2.49) | 0.42 (0.34-0.51) | 1.54 (1.38-1.70) | 1.22 (1.07-1.36) |
| 50-54 | 60 (59-61) | 0.43 (0.34-0.51) | 1.76 (1.59-1.93) | 3.44 (3.20-3.68) | 0.59 (0.49-0.69) | 1.85 (1.68-2.03) | 1.37 (1.22-1.52) |
| 55-59 | 85 (84-86) | 0.48 (0.39-0.58) | 1.60 (1.43-1.77) | 5.27 (4.96-5.57) | 0.83 (0.71-0.95) | 2.23 (2.03-2.42) | 1.61 (1.44-1.77) |
| 60-64 | 105 (103-106) | 0.63 (0.52-0.73) | 1.66 (1.49-1.84) | 6.90 (6.55-7.26) | 1.14 (0.99-1.28) | 2.41 (2.20-2.62) | 1.65 (1.47-1.82) |
| 65-69 | 129 (127-130) | 0.65 (0.54-0.76) | 1.87 (1.68-2.05) | 9.87 (9.44-10.30) | 1.81 (1.63-2.00) | 2.84 (2.61-3.07) | 1.71 (1.53-1.89) |
| 70-74 | 175 (173-177) | 1.05 (0.88-1.23) | 3.01 (2.72-3.31) | 16.83 (16.14-17.53) | 3.78 (3.45-4.11) | 4.97 (4.59-5.35) | 1.91 (1.68-2.15) |
| 75-79 | 238 (235-241) | 1.45 (1.24-1.67) | 4.75 (4.36-5.13) | 27.63 (26.69-28.56) | 7.88 (7.38-8.38) | 7.96 (7.46-8.46) | 2.73 (2.43-3.02) |
| 80-84 | 396 (392-400) | 2.36 (2.07-2.65) | 9.24 (8.67-9.81) | 49.13 (47.81-50.44) | 16.54 (15.78-17.31) | 15.26 (14.53-15.99) | 4.48 (4.09-4.88) |
| 85-89 | 667 (662-673) | 3.33 (2.92-3.74) | 17.30 (16.37-18.23) | 77.70 (75.73-79.67) | 34.14 (32.84-35.45) | 27.90 (26.72-29.08) | 7.04 (6.44-7.63) |
| 90+ | 1037 (1028-1046) | 3.68 (3.12-4.23) | 28.30 (26.76-29.84) | 97.67 (94.81-100.53) | 53.05 (50.94-55.15) | 43.67 (41.76-45.58) | 11.58 (10.60-12.56) |

Supplementary table 5. incidence of fractures by site for women (incidence rate per 10000 patients years with 95 % confidence interval) (continued)

| Age | Proximal upper limb | Distal upper limb | Femoral Neck | Proximal lower limb | Distal lower limb | Multiple fractures |
| --- | --- | --- | --- | --- | --- | --- |
| 20-24 | 1.06 (0.91-1.21) | 17.67 (17.07-18.27) | 0.11 (0.06-0.16) | 0.36 (0.28-0.45) | 9.12 (8.69-9.55) | 2.40 (2.17-2.62) |
| 25-29 | 0.95 (0.82-1.09) | 14.69 (14.16-15.21) | 0.13 (0.08-0.18) | 0.16 (0.11-0.22) | 8.20 (7.81-8.60) | 1.67 (1.49-1.85) |
| 30-34 | 0.86 (0.73-0.98) | 13.22 (12.73-13.71) | 0.13 (0.08-0.18) | 0.17 (0.12-0.23) | 8.12 (7.73-8.50) | 1.28 (1.12-1.43) |
| 35-39 | 0.94 (0.81-1.07) | 13.92 (13.42-14.43) | 0.19 (0.13-0.25) | 0.18 (0.12-0.23) | 9.74 (9.32-10.17) | 1.41 (1.25-1.57) |
| 40-44 | 1.44 (1.29-1.60) | 15.15 (14.64-15.66) | 0.46 (0.37-0.55) | 0.26 (0.20-0.33) | 10.41 (9.99-10.83) | 1.46 (1.30-1.62) |
| 45-49 | 2.15 (1.96-2.34) | 17.21 (16.67-17.75) | 0.91 (0.79-1.04) | 0.41 (0.33-0.49) | 12.31 (11.85-12.76) | 1.86 (1.68-2.03) |
| 50-54 | 3.59 (3.34-3.84) | 26.19 (25.52-26.85) | 2.31 (2.11-2.51) | 0.77 (0.66-0.89) | 15.53 (15.02-16.04) | 2.56 (2.36-2.77) |
| 55-59 | 5.91 (5.59-6.24) | 38.70 (37.88-39.53) | 4.45 (4.17-4.73) | 1.14 (1.00-1.28) | 19.60 (19.02-20.19) | 3.45 (3.21-3.70) |
| 60-64 | 7.96 (7.58-8.34) | 46.84 (45.92-47.77) | 7.52 (7.15-7.89) | 1.81 (1.63-1.99) | 21.80 (21.17-22.43) | 4.32 (4.04-4.60) |
| 65-69 | 11.23 (10.77-11.68) | 53.08 (52.08-54.07) | 12.37 (11.89-12.85) | 3.01 (2.77-3.24) | 23.97 (23.30-24.63) | 6.45 (6.11-6.80) |
| 70-74 | 16.21 (15.53-16.89) | 61.65 (60.32-62.98) | 22.76 (21.95-23.57) | 5.27 (4.88-5.66) | 27.14 (26.26-28.02) | 10.21 (9.67-10.75) |
| 75-79 | 21.28 (20.46-22.10) | 67.47 (66.00-68.93) | 44.72 (43.53-45.92) | 8.84 (8.31-9.37) | 25.69 (24.78-26.59) | 17.73 (16.98-18.48) |
| 80-84 | 31.75 (30.69-32.81) | 89.67 (87.89-91.44) | 93.60 (91.79-95.42) | 18.85 (18.03-19.66) | 29.70 (28.68-30.72) | 35.45 (34.33-36.56) |
| 85-89 | 46.24 (44.72-47.76) | 114.83 (112.44-117.23) | 197.88 (194.73-201.02) | 38.43 (37.05-39.82) | 38.64 (37.25-40.03) | 64.06 (62.27-65.84) |
| 90+ | 63.30 (61.00-65.60) | 135.83 (132.46-139.20) | 376.62 (371.01-382.24) | 74.79 (72.29-77.29) | 51.35 (49.28-53.42) | 97.28 (94.43-100.13) |

Supplementary table 6. Clinical severity of fracture in france in 2016 by gender and site of fractures

|  | Severity | Men  N (%) Mean age (SD) | | Women  N (%) Mean age (SD) | |
| --- | --- | --- | --- | --- | --- |
| All fractures | 1 | 48,998 (20.23) | 43.16 (17.7) | 56,167 (17.56) | 59.16 (19.9) |
|  | 2 | 70,309 (29.03) | 60.72 (21.5) | 93,417 (29.21) | 77.33 (16.6) |
|  | 3 | 116,797 (48.22) | 51.70 (21.4) | 164,493 (51.43) | 71.89 (17.8) |
|  | 4 | 6,132 (2.53) | 75.62 (17.4) | 5,781 (1.81) | 83.70 (12.8) |
| Skull | 1 | NA | NA | NA | NA |
|  | 2 | 2,671 (64.42) | 54.03 (19.8) | 1,294 (70.33) | 67.34 (20) |
|  | 3 | 942 (22.72) | 47.30 (18.9) | 307 (16.68) | 61.50 (19.8) |
|  | 4 | 532 (12.83) | 66.01 (19.1) | 237 (12.88) | 75.99 (16.9) |
| Face | 1 | 245 (1.49) | 36.24 (15.9) | 172 (1.90) | 46.95 (20) |
|  | 2 | 10,259 (62.21) | 46. (21.8) | 6,462 (71.45) | 65.91 (24.2) |
|  | 3 | 5,745 (34.84) | 41.75 (19.3) | 2,268 (25.08) | 59.06 (24) |
|  | 4 | 241 (1.46) | 69.37 (20.6) | 142 (1.57) | 81.66 (14.4) |
| Spine | 1 | NA, | NA | NA | NA |
|  | 2 | 14,548 (70.07) | 68.11 (19.7) | 22,515 (77.58) | 77.86 (15.2) |
|  | 3 | 5,187 (24.98) | 58.37 (18.8) | 5,712 (19.68) | 67.97 (16.5) |
|  | 4 | 1,027 (4.95) | 76.30 (15.3) | 794 (2.74) | 82.51 (12) |
| Pelvis | 1 | NA | NA | NA | NA |
|  | 2 | 3,142 (78.75) | 70.20 (19.4) | 8,922 (92.12) | 81.80 (12.9) |
|  | 3 | 609 (15.26) | 53.12 (21.3) | 520 (5.37) | 68.77 (20.9) |
|  | 4 | 238 (5.96) | 77.42 (16.9) | 243 (2.51) | 84.00 (12.2) |
| Ribs | 1 | NA | NA | NA | NA |
|  | 2 | 13,001 (82.14) | 63.44 (18.2) | 9,231 (87.71) | 75.90 (17) |
|  | 3 | 1,967 (12.43) | 57.59 (18.4) | 868 (8.25) | 67.57 (19.1) |
|  | 4 | 860 (5.43) | 74.39 (16.3) | 426 (4.05) | 80.17 (14.7) |
| Clavicle | 1 | 4,154 (45.77) | 42.94 (17) | 2,367 (48.18) | 57.77 (20.9) |
|  | 2 | 1,973 (21.74) | 57.70 (20.6) | 1,612 (32.81) | 75.96 (17.8) |
|  | 3 | 2,827 (31.15) | 41.19 (15.4) | 866 (17.63) | 52.60 (21.3) |
|  | 4 | 121 (1.33) | 72.21 (20) | 68 (1.38) | 77.38 (18.3) |
| Proximal upper limb | 1 | 1,394 (12.30) | 46.34 (18.6) | 1,608 (7.19) | 61.27 (20.5) |
|  | 2 | 4,121 (36.38) | 64.88 (19.1) | 8,299 (37.11) | 79.11 (13.5) |
|  | 3 | 5,502 (48.57) | 57.75 (18.4) | 12,079 (54.02) | 71.82 (14.3) |
|  | 4 | 312 (2.75) | 75.14 (16.7) | 375 (1.68) | 82.56 (13.3) |
| Distal upper limb | 1 | 31,060 (40.47) | 42.93 (17.9) | 38,645 (40.32) | 60.70 (20) |
|  | 2 | 4,290 (5.59) | 55.52 (23.2) | 8519 (8.89) | 78.18 (16.1) |
|  | 3 | 41,246 (53.74) | 42.67 (17.2) | 48,431 (50.54) | 65.19 (17.1) |
|  | 4 | 160 (0.21) | 74.88 (19.2) | 240 (0.25) | 82.90 (13.1) |
| Femoral neck | 1 | NA | NA | NA | NA |
|  | 2 | 2,058 (9.57) | 76.97 (15) | 4,269 (7.46) | 83.64 (11) |
|  | 3 | 17,991 (83.64) | 77.60 (14.3) | 51,039 (89.14) | 83.41 (10.2) |
|  | 4 | 1,461 (6.79) | 84.24 (10.3) | 1,948 (3.40) | 87.09 (8.9) |
| Proximal lower limb | 1 | 161 (3.26) | 78.17 (14.4) | 418 (3.49) | 83.22 (10.6) |
|  | 2 | 706 (14.28) | 71.04 (18.3) | 1,715 (14.32) | 82.31 (12.8) |
|  | 3 | 3,785 (76.54) | 65.30 (22.4) | 9,428 (78.72) | 81.57 (12.9) |
|  | 4 | 293 (5.93) | 76.36 (17.3) | 416 (3.47) | 85.50 (11.7) |
| Distal lower limb | 1 | 11,982 (30.21) | 43.14 (16.6) | 12,955 (28.83) | 53.93 (18.2) |
|  | 2 | 4,902 (12.36) | 56.67 (20.8) | 7,937 (17.66) | 74.60 (17) |
|  | 3 | 22,597 (56.98) | 46.39 (17) | 23,818 (53.00) | 59.69 (17.5) |
|  | 4 | 178 (0.45) | 70.12 (21.5) | 233 (0.52) | 81.78 (13.8) |
| Multiples fractures | 1 | NA | NA | NA | NA |
|  | 2 | 8,638 (48.68) | 59.07 (20.8) | 12,642 (56.29) | 78.46 (16.2) |
|  | 3 | 8,399 (47.33) | 50.04 (19.8) | 9,157 (40.77) | 73.32 (18.6) |
|  | 4 | 709 (4.00) | 69.18 (20.7) | 659 (2.93) | 81.60 (16.3) |

NA: Not appropriate
